# Phase Demodulation Method for Fringe Projection Measurement Based on Improved Variable-Frequency Coded Patterns

**DOI:** 10.3390/s21134463

**Published:** 2021-06-29

**Authors:** Shanshan Lv, Mingshun Jiang, Chenhui Su, Lei Zhang, Faye Zhang, Qingmei Sui, Lei Jia

**Affiliations:** 1 School of Control Science and Engineering, Shandong University, Jinan 250061, China; lvshanshan@sdu.edu.cn (S.L.); drleizhang@sdu.edu.cn (L.Z.); zhangfaye@sdu.edu.cn (F.Z.); qmsui@sdu.edu.cn (Q.S.); jialei@sdu.edu.cn (L.J.); 2 School of Information and Electrical Engineering, Shandong Jianzhu University, Jinan 250101, China; suchenhui20@sdjzu.edu.cn

**Keywords:** fringe order decoding, VF coded patterns, EWP, FFT

## Abstract

The phase-to-height imaging model, as a three-dimensional (3D) measurement technology, has been commonly applied in fringe projection to assist surface profile measurement, where the efficient and accurate calculation of phase plays a critical role in precise imaging. To deal with multiple extra coded patterns and 2π jump error caused to the existing absolute phase demodulation methods, a novel method of phase demodulation is proposed based on dual variable-frequency (VF) coded patterns. In this paper, the frequency of coded fringe is defined as the number of coded fringes within a single sinusoidal fringe period. First, the effective wrapped phase (EWP) as calculated using the four-step phase shifting method was split into the wrapped phase region with complete period and the wrapped phase region without complete period. Second, the fringe orders in wrapped phase region with complete period were decoded according to the frequency of the VF coded fringes and the continuous characteristic of the fringe order. Notably, the sampling frequency of fast Fourier transform (FFT) was determined by the length of the decoding interval and can be adjusted automatically with the variation in height of the object. Third, the fringe orders in wrapped phase region without complete period were decoded depending on the consistency of fringe orders in the connected region of wrapped phase. Last, phase demodulation was performed. The experimental results were obtained to confirm the effectiveness of the proposed method in the phase demodulation of both discontinuous objects and highly abrupt objects.

## 1. Introduction

Fringe projection technology is a technology that relies on projection device to project sinusoidal patterns onto the surface of the measured object. Then, the morphology of object surface causes deformation to the fringes accordingly. After the deformation image is captured by the camera, the information of the object surface is demodulated by the relevant reconstruction algorithm to perform 3D measurement for the object. Currently, the fringe-projection-based surface profile measurement has been widely used due to advantages such as no contact, high speed, high accuracy, and so on [1,2,3,4]. Among all of the fringe projection techniques, phase calculation plays a crucial role in profile measurement [5]. However, only the wrapped phase ranging from −π to π can be obtained by analyzing the patterns of sinusoidal fringe [6,7,8]. Therefore, phase demodulation is required to obtain absolute phase [9,10]. Traditional phase demodulation techniques include spatial phase unwrapping and temporal phase unwrapping.

Based on the monotonicity of the phase along the direction of fringe projection, the spatial phase unwrapping method can be used to perform phase demodulation by comparing the phase difference between adjacent phases [11,12]. Among the spatial phase unwrapping methods summarized by Ghiglia and Pritt [13], the most popular and effective one is to guide the demodulation path using a quality map. However, due to the impact of noise or varying surface reflectivity, the spatial phase unwrapping method is not completely reliable.

In general, temporal phase unwrapping method relies on auxiliary patterns to make phase unwrapping independent in the time dimension and robust to interference [14,15,16]. Posdamer and Altschuler [17] were the first to propose the use of binary coded patterns for phase demodulation. According to the experimental results, this method can be used to perform real-time measurement directly for thousands of phase points. To reduce the boundary effect while making the system more robust to interference, the gray coding demodulation method was proposed subsequently [18,19]. Due to the interference of object color, inconsistent surface reflectance, and measurement environment, however, there is still no well-developed threshold extraction method, which limits the practical application of the binary and gray coding demodulation methods [20,21,22]. Compared with intensity, the frequency and phase information of fringe are more consistent. Xiao et al. [23,24] proposed to get frequency information integrated into the coded patterns, which is not only independent of the light intensity of the object surface but also conducive to improving the robustness to interference. Since the period of the coded fringe acquired by the camera is modulated by the height of the object [25], however, the accuracy of demodulation results could be affected by the practice of using a fixed sampling frequency to calculate fringe frequency. Zhang et al. [26] proposed the use of phase as coding information to perform phase demodulation and then carried out experiments to verify the feasibility and stability of the phase coding demodulation at different degrees of exposure. In order to improve the maximum decoding fringe orders, Da et al. [27] integrated the coded phase into two groups of sinusoidal fringes with varying frequency. To reduce the number of coded patterns needing to be projected while speeding up the measurement, Chen et al. [28] designed the phase information as a specific coding sequence and determined the fringe orders by comparing the position of the coding phase in the coding sequence. In addition, the sinusoidal patterns can also be used as auxiliary coded patterns to decode fringe orders. As the most commonly used temporal phase unwrapping method at present, the multifrequency phase-shift method determines the absolute phase by comparing the wrapped phase under different fringe frequency as obtained by the phase shifting algorithm [29,30,31]. Though the temporal phase unwrapping methods as described above can achieve higher measurement accuracy, the measurement of the system tends to be slowed down if there are too many coded images to be projected.

Most recently, a novel phase demodulation method has been proposed on the basis of multiview geometric constraints by increasing the number of cameras [32,33,34]. According to this method, wrapped phase, epipolar geometry, or phase monotonicity is usually treated as the constraint on phase demodulation. Since the projector can be regarded as a reverse camera in the measurement system, An et al. [35] presented a phase demodulation method according to the minimum absolute phase map $\Phi_{\min}$. Without increasing the number of cameras, the fringe order in each pixel can also be determined based on the geometric constraints of the system. However, the measurement range that applies to the method proposed by An et al. [35] is restricted to between the minimum plane $Z_{\min}$ and the plane causing 2π phase change; otherwise, decoding error will arise. Jiang et al. [36] overcame the limitations on this method by building multiple minimum absolute phase maps and performing phase demodulation in different regions through the use of prior knowledge. Though the method based on geometric constraints reduces the time required for image projection and acquisition, it requires the system parameters to be precalibrated, and the accuracy of phase demodulation will be affected by the results of system calibration. Moreover, the demodulating area is determined by the common viewing angle of camera and projector, which makes it unsuited to the measurement of large objects.

Given the problem that the measurement can be slowed down by too many coded patterns to be projected when the temporal phase unwrapping method is used, a phase demodulation method based on dual VF coded patterns is proposed. In this paper, there are two groups of fringe patterns to be projected. The regular spaced sinusoidal fringe patterns are used to obtain the wrapped phase and the coded fringe patterns are used to number the sinusoidal fringe (i.e., determine the fringe orders), whose frequency is higher than that of the sinusoidal fringe. In the process of fringe orders decoding, the coded fringe of each row is split into several groups according to the wrapped phase. Additionally, the sampling frequency of FFT is determined by the element numbers of the decoding interval, rather than a constant. Therefore, the proposed phase demodulation method remains effective even though the object surface is discontinuous or there is abrupt height variation.

## 2. Encoding and Decoding Principle of the Dual VF Coded Patterns

### 2.1. Encoding Principle

The frequency-based coded pattern is characterized by anti-interference to light intensity. The intensity ($c_{1}$ and $c_{2}$) of the two coded patterns (VF1 and VF2) projected is expressed as Equation (1).

(1) $$\begin{array}{c} c_{1}\left(u,v\right)=a+b\cdot \cos\left[2\pi \cdot \frac{\mathrm{mod}\left(\left\lfloor u/T\right\rfloor ,f_{h}\right)+1}{T}\cdot u\right]\\ c_{2}\left(u,v\right)=a+b\cdot \cos\left[2\pi \cdot \frac{\mathrm{mod}\left(\left\lfloor u/\left(f_{h}\cdot T\right)\right\rfloor ,f_{h}\right)+1}{T}\cdot u\right] \end{array}$$

where $\left(u,v\right)$ denotes the pixel coordinate; *a* and *b* refer to the average and the modulated intensity, respectively; T indicates the period of the sinusoidal fringe; $f_{h}$ stands for the maximum frequency of the coded fringe in one period; $\left\lfloor \cdot \right\rfloor$ represents the operation of rounding down; and the function mod $\left(x,y\right)$ refers to the remainder of *x* divided by *y*. According to Equation (1), the frequency-change period of VF1 is T, which is the same as the period of the sinusoidal fringe. The frequency-change period of VF2 is $f_{h}\cdot T$, which is $f_{h}$ times VF1. That is to say, VF2 can be regarded as the orders encoding of VF1.

**Figure 1:**
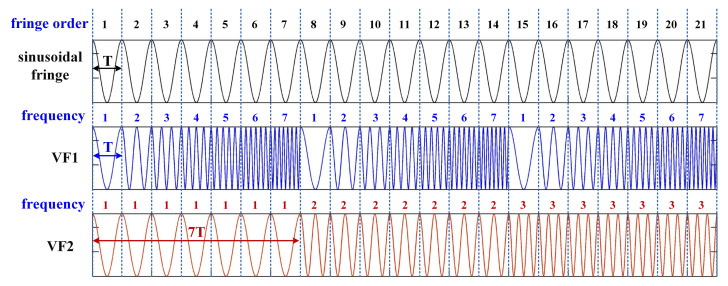
Sinusoidal and coded fringes of the first 21 sinusoidal periods, in which T is the period of the sinusoidal fringe.

Given the accuracy of frequency recognition, $f_{h}$ is set to 7 in this paper. In order to better show the periodic variation of the dual VF patterns, only the first 21 periods of the coded fringes are shown in Figure 1.

### 2.2. Decoding Principle

Modulated by the surface of the object, the period of sinusoidal fringes collected by the camera is not necessarily complete. For the complete-period fringes, FFT is adopted to calculate the fringe order. Unlike conventional FFT algorithm, the sampling frequency of FFT is set as equal to the number of pixels in the current decoding interval for obtaining the number of periodic variations of the coded fringes in the decoding interval. Subsequently, the order of complete-period fringes can be obtained using Equation (2).

(2) $$\begin{array}{c} n=f_{1}+\left(f_{2}-1\right)\cdot f_{h} \end{array}$$

In Equation (2), *n* represents the fringe order; $f_{1}$ and $f_{2}$ denote the frequency of VF1 and VF2, respectively.

**Figure 2:**
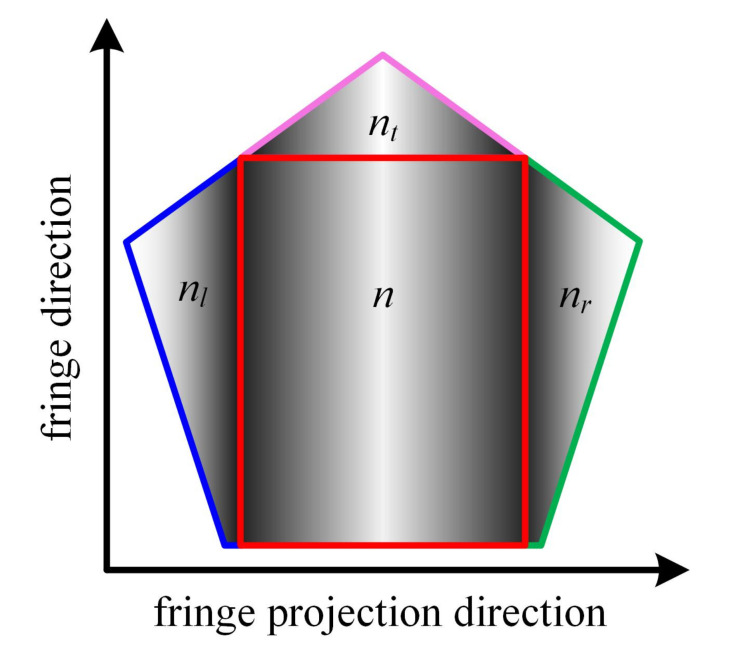
Partial sinusoidal fringe image acquired by camera.

As for the incomplete-period fringes, orders decoding can be performed according to the continuity of orders in the direction of fringe projection and consistency in the direction of fringe. As shown in Figure 2, it is assumed that the order of the complete-period fringe in the red rectangle is *n*, the order of the incomplete-period fringe in the green rectangle is $n_{r}=n+1$, and the order of the incomplete-period fringe in the blue rectangle is $n_{l}=n-1$. The sinusoidal fringes in the pink rectangle and the red rectangle fall within the same period, so that $n_{t}=n$.

## 3. Application of Dual VF Coded Patterns in Phase Demodulation

**Figure 3:**
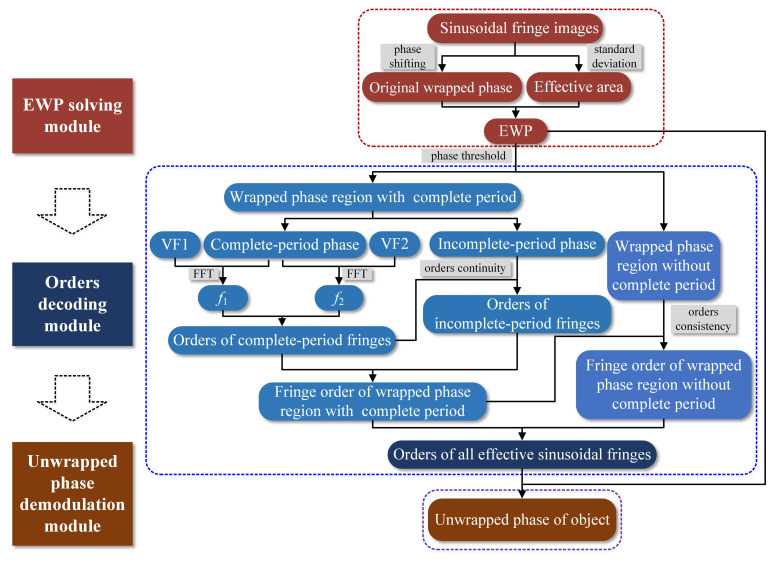
Flow chart of the effective phase unwrapping.

As shown in Figure 3, the absolute phase solving process involves three sections, including the EWP solving module, orders decoding module, and unwrapped phase determination module. First of all, the wrapped phase of the object (i.e., EWP) is determined by the phase shifting algorithm and the standard deviation of the sinusoidal patterns. Then, FFT algorithm is combined with the continuity and consistency of fringe orders, while the orders of all effective sinusoidal fringes are decoded. Last, the unwrapped phase map of the object is built.

### 3.1. EWP Calculation

In the four-step phase shifting algorithm [37], the intensity of sinusoidal fringe images as captured by camera is expressed as:

(3) $$\begin{array}{c} I_{i}=I^{\prime }+I^{\prime \prime }\cdot \cos\left(\Phi -\frac{3\pi }{2}+\frac{\pi }{2}i\right),i=1,2,3,4 \end{array}$$

where $I_{i}$ represents the reflected intensities, $I^{\prime }$ indicates the background intensity, $I^{\prime \prime }$ denotes the modulated intensity, and Φ refers to the modulated phase associated with the height of the object. Based on the trigonometric function algorithm, the original wrapped phase $\Phi_{o}$ of the detected area is calculated using Equation (4).

(4) $$\begin{array}{c} \Phi_{o}=\arctan\frac{I_{4}-I_{2}}{I_{1}-I_{3}} \end{array}$$

Due to the measured background or shadows, it is common for the invalid phase to exist in the original wrapped phase $\Phi_{o}$. In this paper, the invalid area refers to the shadow or strong reflective area, whose light intensity of this area is approximately 0 or reaches the maximum value, which is close to saturation. Moreover, the light intensity will not change under the projection of sinusoidal fringe patterns with different initial phases, so the light intensity standard deviation in invalid area is close to 0. Since the intensity change of sinusoidal fringes in the invalid area is far smaller than that of fringes on the surface of the object [38], the intensity standard deviation of the sinusoidal patterns is defined as Equation (5) to remove the invalid phase.

(5) $$\begin{array}{c} S=\sqrt{\frac{1}{4}\sum {\left(I_{i}-\bar{I}\right)}^{2}} \end{array}$$

In Equation (5), $\bar{I}$ denotes the average intensity of sinusoidal fringes, which is expressed as Equation (6).

(6) $$\begin{array}{c} \bar{I}=\frac{1}{4}\sum I_{i} \end{array}$$

Then, to define a flag matrix G, each element g(m,n) of the matrix G is defined as follows.

(7) $$\begin{array}{cc} g\left(m,n\right)=1, & \mathrm{if}\;s\left(m,n\right)\geq s_{h}\\ g\left(m,n\right)=0, & \mathrm{if}\;s\left(m,n\right)<s_{h} \end{array}$$

In Equation (7), the parameter $s\left(m,n\right)$ denotes the intensity standard deviation at the pixel coordinate $\left(m,n\right)$; $s_{h}$ indicates the intensity threshold for binarization, which is artificially set based on the result of light intensity standard deviation ($s_{h}$ was set to 0.005 in this paper). After this step, the region with element 1 in G is treated as the effective measurement region.

Last, EWP $\Phi_{e}$ is calculated using Equation (8), while the arithmetic symbol ·∗ represents an operator multiplication of elements in different matrices but in the same position.

(8) $$\begin{array}{c} \Phi_{e}=\Phi_{o}\cdot *G \end{array}$$

### 3.2. Orders Decoding with Dual VF Coded Patterns

**Figure 4:**
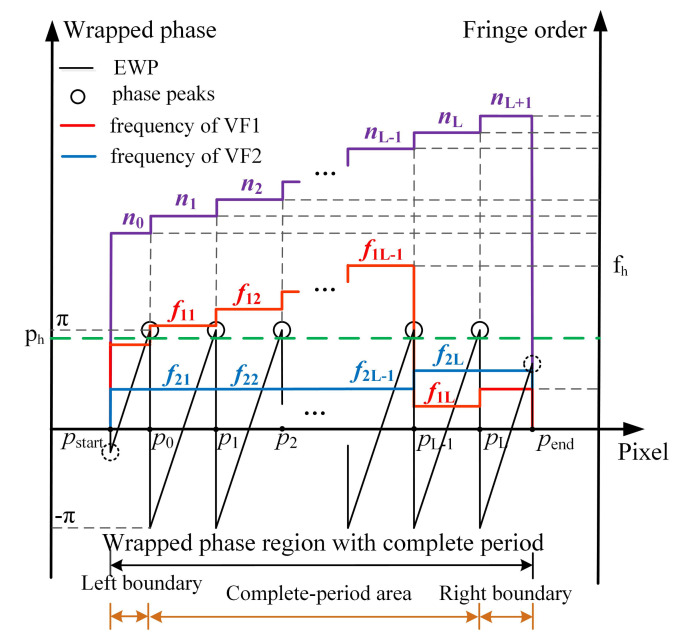
Orders decoding of wrapped phase with complete period.

**Figure 5:**
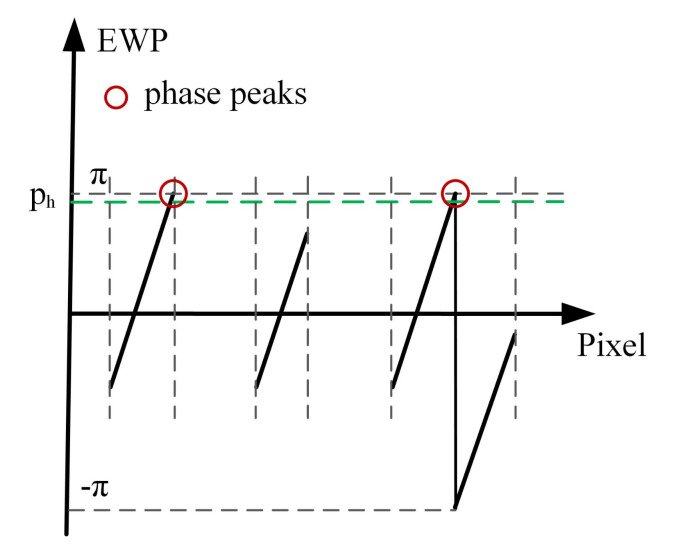
Wrapped phase without complete period.

**Figure 6:**
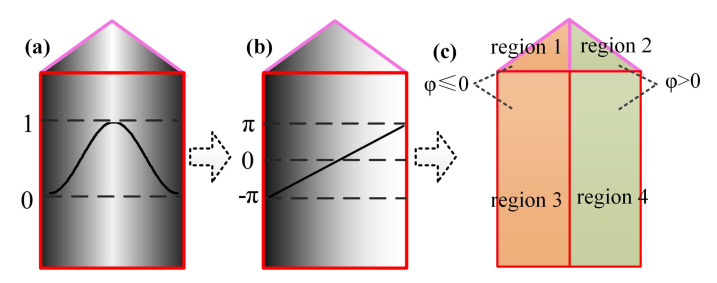
Recognition of semiperiodic connected regions of EWP: (**a**) sinusoidal fringe; (**b**) EWP; (**c**) semiperiodic wrapped phase connected regions, φ is the wrapped phase.

Take any row of wrapped phase as an example. According to the corresponding relationship between fringe order and frequency of coded fringe, the process of orders decoding detailed as follows:

**Step 1.**  Initialization is carried out. The fringe order set n is initialized to 0.
**Step 2.**  EWP classification is performed. On the whole, the period of the sinusoidal fringes projected on the object is not necessarily complete. Accordingly, by setting a phase threshold $p_{h}$ to be slightly less than π, all phase peaks with phase greater than $p_{h}$ are recorded. The region containing at least two phase peaks is defined as the wrapped phase region with complete period (Figure 4). Otherwise, it is the wrapped phase region without complete period (Figure 5).
**Step 3.**  The wrapped phase region with complete period is further divided into complete-period phase region and boundary phase region (left boundary and right boundary). As shown in Figure 4, the phase peak index set is $P=\left[p_{0},p_{1},\;\cdots ,p_{L}\right]$, of which the wrapped phase value is higher than $p_{h}$. It is defined that the phase located in pixel interval $\left[p_{i-1},p_{i}\right]$$\left(i=1,\;\cdots ,L\right)$ is the complete-period phase, and the pixel interval $\left[p_{0},p_{L}\right]$ is the complete-period phase region. The pixel interval $\left[p_{\mathrm{start}},p_{0}\right]$ is taken as the left boundary phase region, the pixel interval $\left[p_{L},p_{\mathrm{end}}\right]$ is treated as the right boundary phase region, and the wrapped phase located at the boundary phase region is classed as the boundary phase.
**Step 4.**  The orders decoding of the fringes corresponding to the complete-period phase is performed. According to the description made in Section 2.2, the frequency ($f_{1i}$ and $f_{2i}$) of the two VF fringes (VF1 and VF2) in each pixel interval $\left[p_{i-1},p_{i}\right]$ can be calculated. Then, the orders $\left[n_{1},n_{2},\;\cdots ,n_{L}\right]$ of the fringes corresponding to complete-period phase are obtained using the formula $n_{i}=f_{1i}+\left(f_{2i}-1\right)\cdot f_{h}$.
**Step 5.**  The orders decoding of the fringes corresponding to boundary phase is performed. According to the continuity of the fringe orders in the direction of fringe projection, the order of the fringe corresponding to left boundary phase is $n_{0}=n_{1}-1$, and the order of the fringe corresponding to right boundary is $n_{L+1}=n_{L}+1$. Given the whole wrapped phase region with complete period, the corresponding fringe orders can be expressed as $n=[n_{0},n_{1},\;\cdots ,n_{L},n_{L+1}]$.
**Step 6.**  The orders decoding of the fringes corresponding to the wrapped phase without complete period is performed. When the number of phase peaks above the phase threshold $p_{h}$ is less than 2, it is considered that there is no sinusoidal fringe with complete period in the current decoding period, as shown in Figure 5. To determine the fringe orders corresponding to the incomplete-period wrapped phase, all connected regions with phase greater than 0 and less than or equal to 0 are identified to identify the semiperiodic connected regions of wrapped phase (Figure 6). Since the sinusoidal fringes in the same connected region fall within the same period, the fringe orders located in the same phase connected region are equal. As shown in Figure 6, the fringe orders of region 1 are equal to that of region 3, while the fringe orders of region 2 are equal to that of region 4. Therefore, the fringe orders corresponding to the wrapped phase region without complete period can be determined according to that of complete period.

In case that the surface of the object is discontinuous or there are multiple objects in the measurement area, it is possible for some phase peaks to be missed. In this circumstance, the EWP is first split into several subeffective EWP areas, and then the fringe order decoding is performed by following the above steps.

### 3.3. Unwrapped Phase Demodulation

Through a combination between the EWP and fringe orders [39], the absolute phase can be demodulated using Equation (9).

(9) $$\begin{array}{c} \Phi_{D}=\Phi_{e}+2\pi \cdot N \end{array}$$

In Equation (9), N represents fringe order of the effective measurement area; $\Phi_{D}$ refers to the demodulated phase.

## 4. Experiment and Analysis

In this section, the experimental system is constructed to carry out phase unwrapping experiments on different objects, so as to verify the proposed phase demodulation method for its feasibility and accuracy.

### 4.1. Experimental System Construction

**Figure 7:**
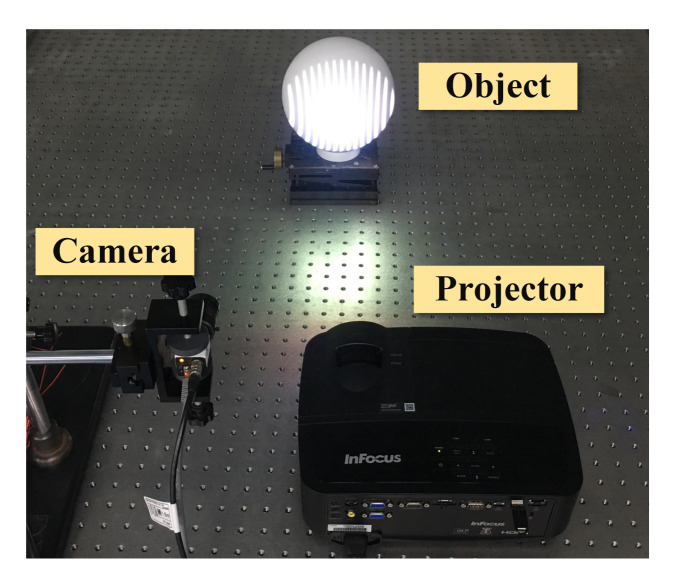
Experimental system.

Figure 7 shows the experimental system constructed, where the DLP projector (InFocus, IN2128HDx, Portland, OR, USA) with a resolution of 1920 × 1080 was employed to project sinusoidal and coded patterns, while the black-and-white CCD camera (DAHENG, MER-500-14GM, Beijing, China) with a resolution of 2592 × 1944 was applied to capture the modulated images on the object surface. Then, the captured images were transmitted to the computer via the Gigabit Ethernet line for subsequent processing and analysis.

**Figure 8:**
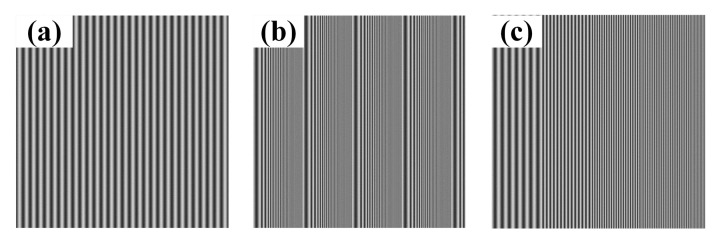
The computer-generated pattern: (**a**) sinusoidal fringe pattern with initial phase of −π; (**b**) coded fringe pattern VF1; (**c**) coded fringe pattern VF2.

The sinusoidal and coded patterns are generated on the computer using MATLAB, as shown in Figure 8. To be specific, the sinusoidal fringe pattern projected by the projector has a size of 1920 pixel × 1080 pixel and a period of 64 pixel. There are 30 periods in one projected sinusoidal fringe pattern. The size of the coded patterns is the same as that of the sinusoidal fringe pattern, i.e., 1920 pixel × 1080 pixel. The frequency of VF1 and VF2 is changing from 1 to 7 and from 1 to 5, respectively. Even if calculation is performed according to the maximum frequency (*f* = 7) of coded fringe, each complete coded fringe projected by the projector contains at least 9 (64/7) discrete points, which basically meets the requirement of 10× sampling. Moreover, the transverse resolution of camera is higher as compared to the projector, so that the camera part also approximately meets the 10× sampling requirement. On the whole, according to the sampling theorem, the accuracy of fringe orders decoding can be ensured when FFT is applied to calculate the frequency of coded fringes.

### 4.2. Phase Unwrapping of Surface Continuous Object

**Figure 9:**
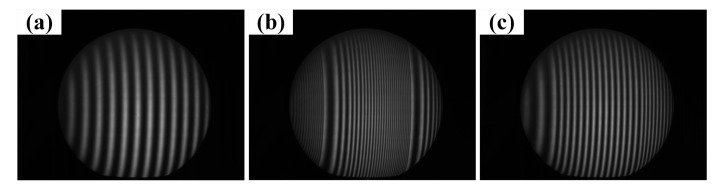
Images captured by the camera: (**a**) sinusoidal fringe image with initial phase of −π; (**b**) coded image VF1; (**c**) coded image VF2.

A solid gypsum sphere with the diameter of 15 cm is exemplified to discuss the application of the proposed method. Figure 9 shows the sinusoidal and coded images of the sphere surface as captured by the camera.

**Figure 10:**
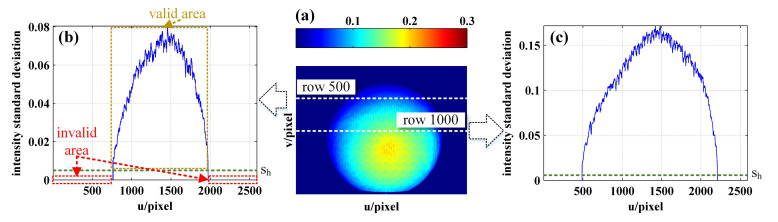
(**a**) Intensity standard deviation map; (**b**) the intensity standard deviation of row 500; (**c**) the intensity standard deviation of row 1000.

**Figure 11:**
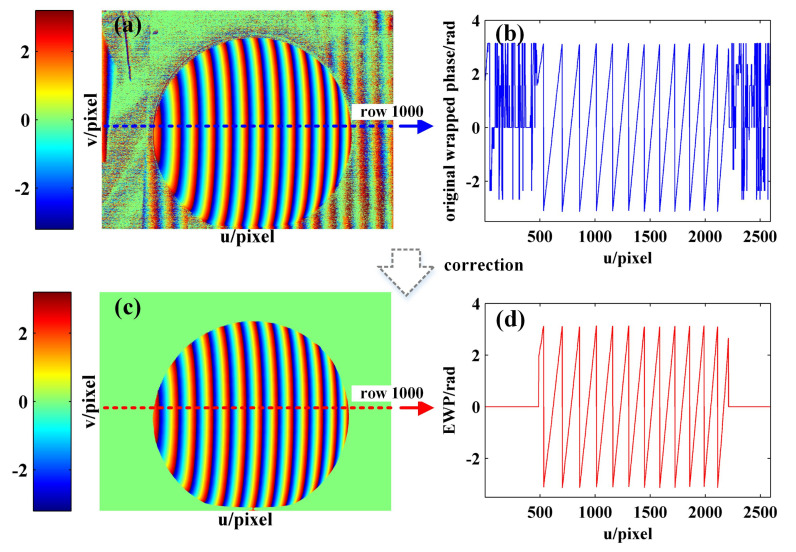
(**a**) Original wrapped phase map; (**b**) original wrapped phase of row 1000; (**c**) EWP map; (**d**) EWP of row 1000.

First, the EWP map is built. According to the sinusoidal images and Equation (5), the intensity standard deviation map is calculated, as shown in Figure 10a. From Figure 10b,c, it can be seen that the intensity standard deviation of the sphere surface is significantly larger than that of the invalid area, as a result of which the invalid wrapped phase can be recognized and removed by setting the intensity standard deviation threshold $s_{h}$ (the green line in Figure 10b,c). Figure 11a,b show the original wrapped phase map and original wrapped phase of row 1000 as calculated according to the four-step phase-shifting method, respectively, while Figure 11c,d show the EWP map and the EWP of row 1000 as obtained according to the light intensity standard deviation correction, respectively.

**Figure 12:**
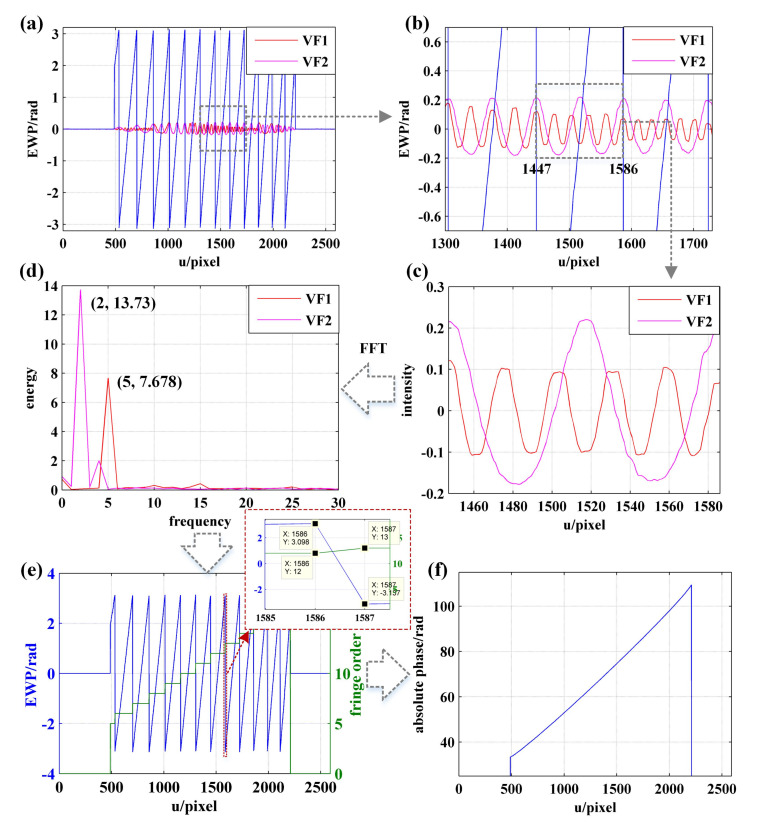
Phase unwrapping result of row 1000 obtained using the proposed method: (**a**) EWP and coded fringes; (**b**) EWP and coded fringes with pixel interval of [1300, 1730]; (**c**) coded fringes with pixel interval of [1447, 1586]; (**d**) FFT spectrum of (c); (**e**) EWP and fringe order; (**f**) absolute phase.

Then, the fringe order with complete period is calculated using FFT. For example, Figure 12a shows the EWP and VF fringes of row 1000. As can be seen from the enlarged image (Figure 12b), the pixel interval [1447, 1586] is a complete fringe period. Therefore, the sampling frequency should be set to 140 when FFT is used to solve the fringe order of the current period. Figure 12d presents the FFT spectrum of Figure 12c, from which it can be seen that the frequency of VF1 and VF2 in interval [1447, 1586] is 5 and 2, respectively. According to Equation (2), the fringe order in interval [1447, 2586] is 5+(2−1)·7=12. Then, the order of remaining fringes with complete period is also determined using the same method described above. Last, the order of nonholonomic periodic fringe is determined according to the continuity and consistency of fringe order. According to the enlarged image of fringe order shown in Figure 12e, it can be seen that the fringe order obtained using the proposed method is consistent with the jump position of the wrapped phase. As a result, at the junction of fringes with different periods, there is no 2π jump error existed in absolute phase obtained using the proposed method, as shown in Figure 12f.

**Figure 13:**
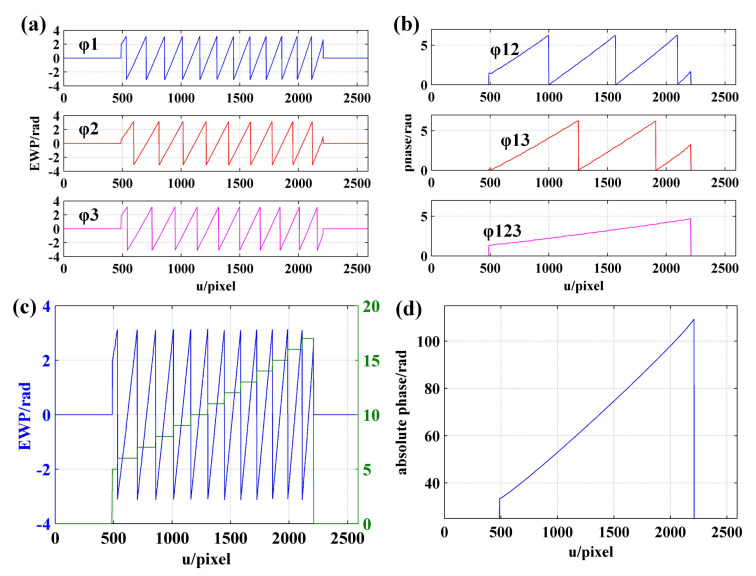
Phase unwrapping result of row 1000 obtained by three-frequency phase-shift method: (**a**) EWP; (**b**) equivalent phase; (**c**) EWP and fringe order; (**d**) absolute phase.

In order to verify the accuracy of the proposed method, the fringe order map and the unwrapped phase map obtained using the multifrequency phase-shift method [40] are taken as reference for comparison against the proposed method. In this paper, the periods of sinusoidal fringe patterns generated for the three-frequency phase-shift method are T1 = 64 pixels, T2 = 85 pixels and T3 = 80 pixels, respectively. Figure 13a shows the EWP of the row 1000 on the sphere surface corresponding to the different frequencies as obtained using the phase-shifting algorithm, and Figure 13b shows the equivalent phase. Figure 13c,d show the fringe order and absolute phase with a corresponding period of 64 pixel as obtained using the multifrequency phase-shift method. Compared with Figure 12 and Figure 13, the results of fringe order as obtained using the two methods are consistent, suggesting that the proposed method is feasible and accurate in decoding fringe order.

**Figure 14:**
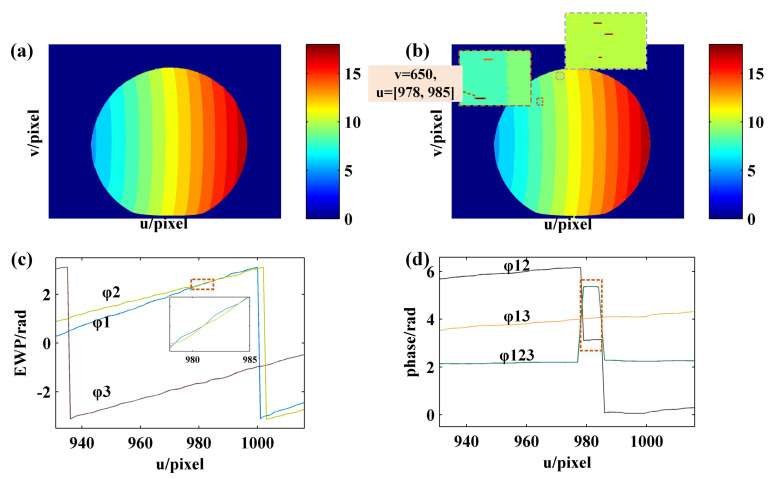
Comparison results of the sphere fringe orders: (**a**) fringe order map obtained by proposed method; (**b**) fringe order map obtained by three-frequency phase-shift method; (**c**,**d**) are, respectively, the EWP and equivalent phase of row 650 calculated for three-frequency phase-shift method.

As suggested by the comparison results shown in Figure 14, the method proposed in this study can be applied to obtain the same accuracy as the multifrequency phase-shift method and there are as few as two coded images required. Moreover, Figure 14 reveals that the existence of phase nonlinear errors may render the demodulation results of the multifrequency phase-shift algorithm incorrect at the interface of EWP with different frequencies. In comparison, the proposed method relies on the frequency information of the coded fringes, which requires low accuracy of the wrapped phase.

### 4.3. Phase Unwrapping of Surface Discontinuous Object

**Figure 15:**
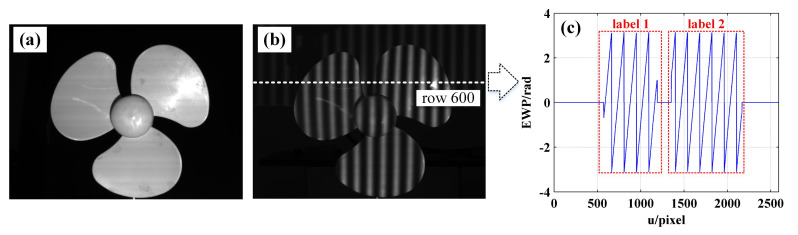
(**a**) Physical map of fan blade; (**b**) sinusoidal fringe image with initial phase of −π captured by camera; (**c**) EWP of row 600.

In order to verify the feasibility of the proposed method on surface discontinuous objects, the phase unwrapping experiment of the blue three-blade fan was carried out. Figure 15a shows the physical map of the fan blade, and Figure 15b shows the sinusoidal fringe image with an initial phase of −π as captured by the camera. Since the surface of the object is discontinuous, there is a possibility that the EWP of a certain row is discontinuous, as shown in Figure 15c. In this circumstance, the EWP of row 600 can be split into two subeffective regions in the first place (label 1 and label 2).

**Figure 16:**
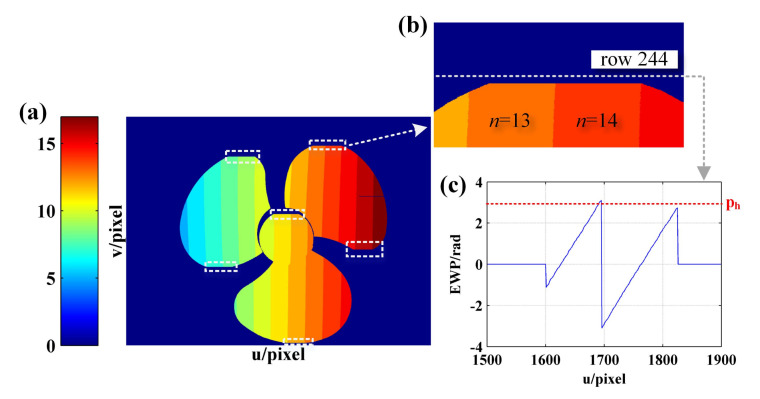
(**a**) The preliminary phase order map; (**b**) partial EWP with u = [1500, 1900] and v = [150, 350]; (**c**) EWP with u = [1500, 1900] of row 244.

Then, according to Steps 3–5 in the decoding process as detailed in Section 3.2, the fringe order in regions with complete period is decoded and the preliminary fringe order map shown in Figure 16a is obtained. In Figure 16a, the proportion of the white rectangular boxes in which the fringe order is left not decoded represents the regions without complete period, as shown in Figure 16b,c, respectively.

**Figure 17:**
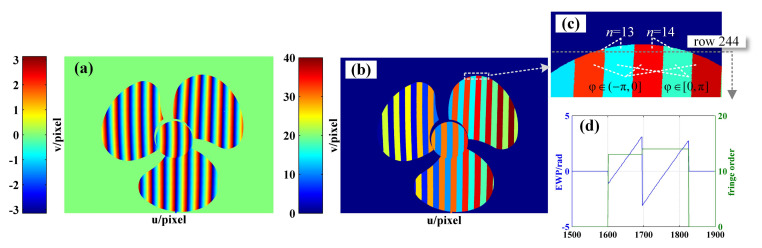
(**a**) EWP map; (**b**) identification result of semiperiodic connected regions; (**c**) enlarge view of (**b**) with u = [1500, 1900] and v = [150, 350]; (**d**) EWP and fringe order with u = [1500, 1900] of row 244 obtained using the proposed method.

Then, in order to perform fringe order decoding for the regions without complete period, the semiperiodic connected region of EWP is identified according to Step 6 as detailed in Section 3.2. The identification result is shown in Figure 17b, where the regions of the same color fall within the same connected region. When the phase difference of the adjacent pixels caused by the height change of the object surface is in no excess of 2π, the fringe order falling into the same connected region is equal. According to this property, the fringe order of the regions without complete period as shown in Figure 16a can be decoded, while the results are shown in Figure 17c,d, respectively.

**Figure 18:**
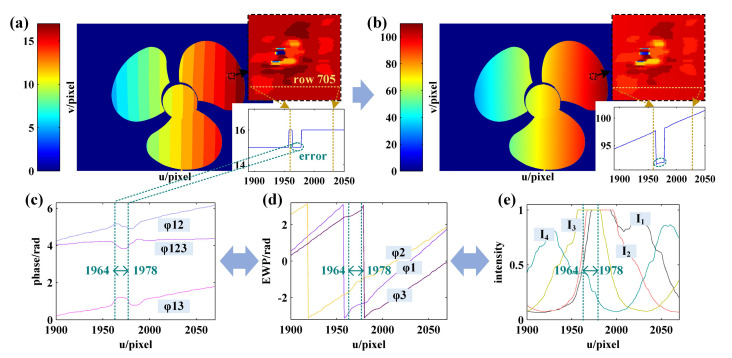
Phase unwrapping results of the three-blade fan obtained by three-frequency phase-shift method: (**a**) fringe order map; (**b**) absolute phase map; (**c**–**e**) are the equivalent phase, EWP, and sinusoidal fringe of row 705, respectively.

**Figure 19:**
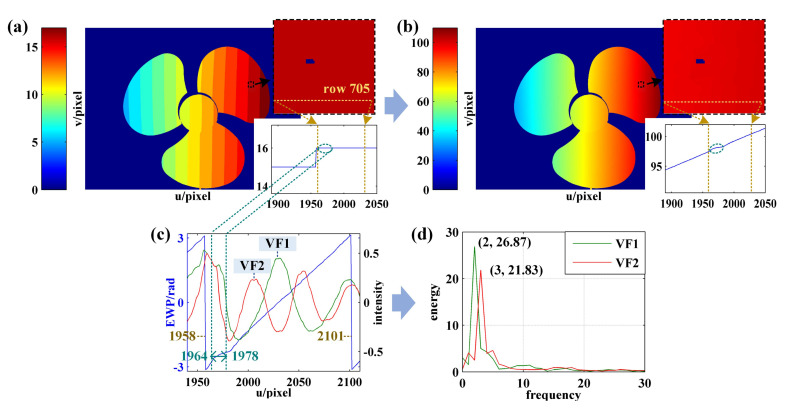
Phase unwrapping results of the three-blade fan obtained using the proposed method: (**a**) fringe order map; (**b**) absolute phase map; (**c**) EWP and coded fringes of row 705; (**d**) FFT spectrum of coded fringes in pixel interval [1958 2101] of row 705.

Figure 18 and Figure 19 present the phase unwrapping results of the fan blade obtained by three-frequency phase-shift and proposed methods. By comparing Figure 18a,b and Figure 19a,b, it can be seen that the results obtained using the two methods are highly consistent except for the area marked by black rectangle. According to the enlarged image shown in Figure 18a,b, there are some errors in the fringe order and absolute phase obtained by the three-frequency phase-shift method. Take row 705 as an example. The fringe order in pixel interval [1964, 1978] should be decoded as 16, while the result as obtained using the three-frequency phase-shift method is 15. According to the three-frequency phase-shift method, it is known through analysis that the error might be attributed to the reflection characteristic of the object surface. As shown in Figure 18e, the light intensity of some sinusoidal fringes near the pixel interval [1964, 1978] reaches its maximum. In this circumstance, the calculated EWP (Figure 18d) is inaccurate, as is the further calculated equivalent phase (Figure 18c), thus resulting in the wrong fringe order.

The accurate results can be obtained using the proposed method, which is pontentially because the fringe order in the pixel interval [1964, 1978] is determined by the coded fringe frequency in the complete period interval [1958, 2101]. Figure 19d presents the FFT spectrum of coded fringes in pixel interval [1958, 2101]. It can be obtained that $f_{1}$ = 2 and $f_{2}$ = 3. Then, according to Equation (2), the fringe order in pixel interval [1958, 2101] can be obtained as 16. Based on the above analysis, the implementation of three-frequency phase-shift algorithm depends on the phase calculated according to multifrequency fringes. Therefore, the fringe order will be incorrect when the calculated phase is wrong. The implementation of the proposed method is dependent on the frequency of the coded fringe. To sum up, the accurate fringe order is achievable as long as the frequency of the fringe is unaffected.

### 4.4. Phase Demodulation of the Object with Height Abrupt Change

**Figure 20:**
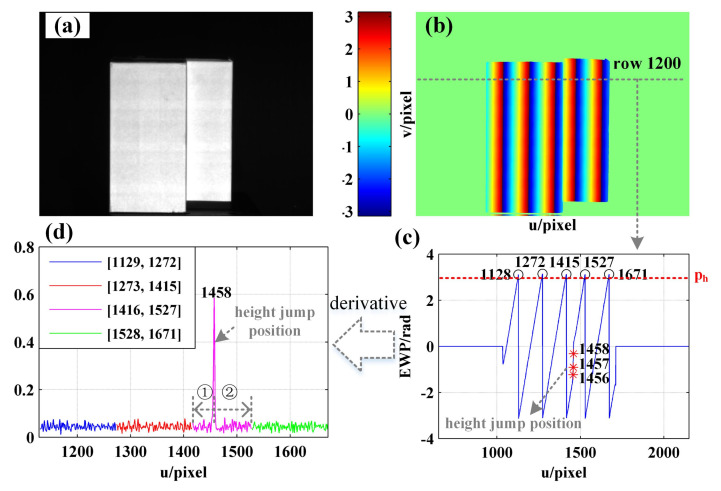
(**a**) Physical map of the step workpiece; (**b**) EWP map with u = [650, 2150] and v = [800,1920]; (**c**) EWP with u = [650, 2150] of row 1200; (**d**) the derivative of (**c**).

In order to further verify the accuracy of the proposed method on the objects with abrupt change to height, another experiment is performed on a step workpiece as shown in Figure 20a,b shows the EWP as obtained using the phase-shifting algorithm and intensity standard deviation, while Figure 19c shows the EWP of row 1200. As shown in Figure 20c, 1128, 1272, 1415, 1527, and 1671 represent the positions in which the wrapped phase peak is higher than $p_{h}$, with the change to height of the object being around 1456–1458 pixels. The phase derivative results of adjacent phase peak interval [1129, 1272], [1273, 1415], [1416, 1527], and [1528, 1671] are obtained, as shown in Figure 20d, from which it can be seen that the phase derivative in the position where the height of the object changes suddenly is significantly higher than in other positions. Therefore, the phase derivative curve can be used to determine whether the height of the object changes abruptly in the current decoding period. Then, the decoding period containing the object height change position is divided into two parts (i.e., ① and ② in Figure 20d), and phase unwrapping is performed using the boundary fringe order decoding method.

**Figure 21:**
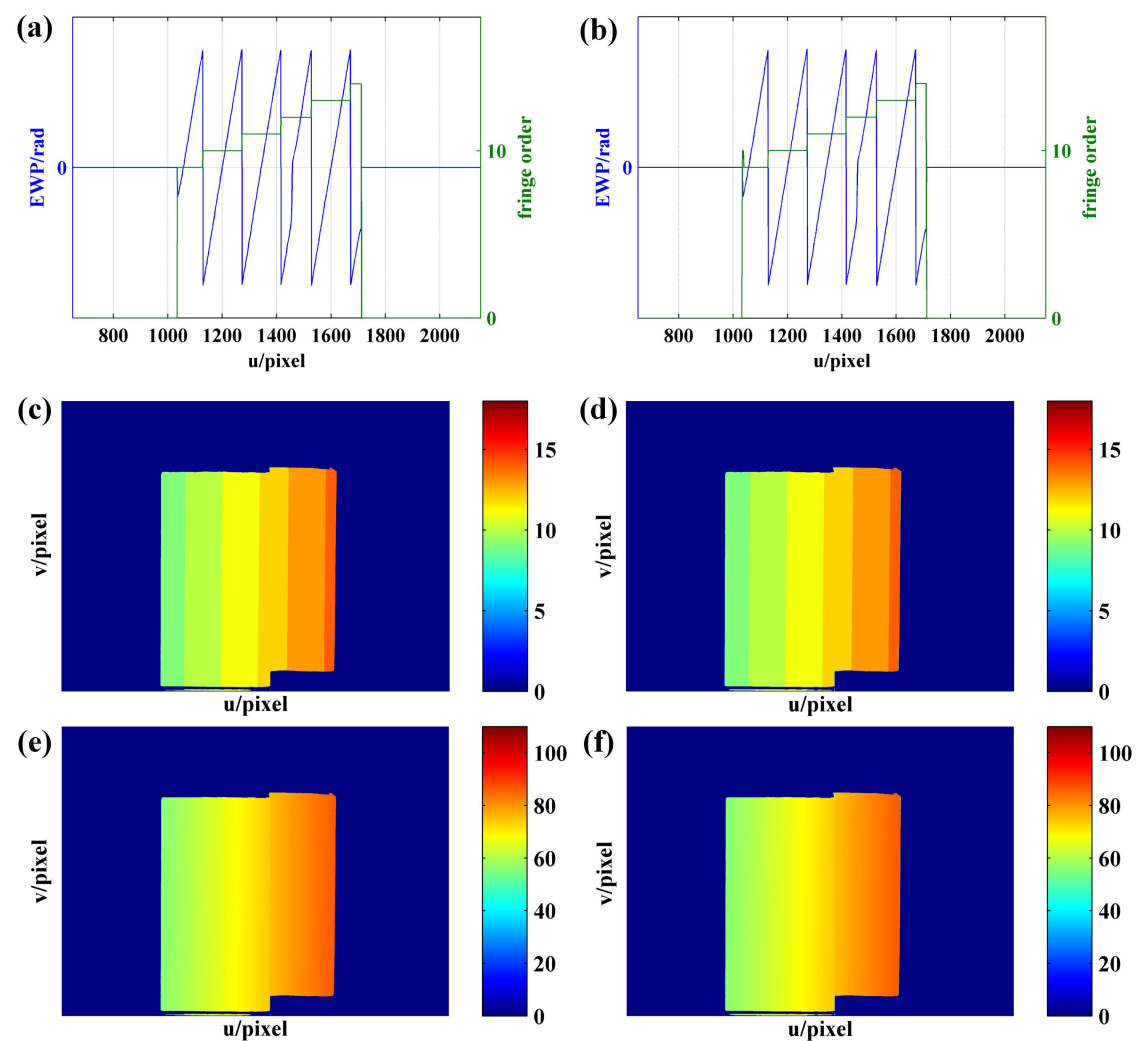
Phase unwrapping results of the step workpiece: (**a**,**c**,**e**) are the fringe order with u = [650, 2150] of row 1000, fringe order map, and unwrapped phase obtained using the proposed method, respectively; (**b**,**d**,**f**) are the fringe order with u = [650, 2150] of row 1000, fringe order map, and unwrapped phase obtained using the three-frequency phase-shift method, respectively.

As suggested by the phase unwrapping results of the step workpiece as shown in Figure 21, the proposed method is applicable to the phase unwrapping of objects with abrupt change to height. The accuracy of demodulation can reach the same level as when the three-frequency phase-shift method is used.

For the proposed method and the three-frequency phase-shift method, two programs were written using MATLAB to perform phase unwrapping based on the acquired fringe images. The average of ten running times of the two methods is calculated, and the result is shown in Table 1.

As shown in Table 1, shorter running time is needed for the three-frequency phase-shift method, and the time-consuming difference is not significant for different objects. Since the proposed method decodes the fringe orders row by row and calls a large number of functions, it takes longer time than the three-frequency phase-shift method. Moreover, the more complex geometry of the object, the more running time is required. In the follow-up study, the running time could be shortened by optimizing the program.

## 5. Conclusions

In this study, a novel phase demodulation method developed on the basis of improved VF coded patterns is proposed for the fringe projection system. An introduction is made of the design of the coded patterns and the principle of phase demodulation. This method demonstrates the following advantages:

1. Throughout the phase demodulation process, there are as few as two additional VF coded patterns required except for the sinusoidal patterns used to determine the wrapped phase, which is conducive to reducing the number of projected patterns and making the measurement process easier to carry out.
2. Since the two coded fringes are decoded in the same interval as the wrapped phase, the phase jump error of 2π is eliminated from the absolute phase demodulated using the proposed method. Thus, it is needless to perform the subsequent phase filtering or fringe orders correction operation.
3. In respect of orders decoding, the sampling frequency of FFT algorithm is equal to the length of the current decoding interval, which can be adjusted automatically depending on the surface variation of the object. Thus, this method can be applied to achieve phase demodulation for both smooth surface objects and the objects with abrupt height change.

In order to demonstrate the demodulation performance of VF patterns, the comparative experiment is carried out between the proposed method and the multifrequency phase-shift method. According to the experimental results, the proposed method is effective in achieving the same accuracy as the multifrequency phase-shift method, so that there are fewer images required.

## 6. Discussion

The frequency-coding-based phase unwrapping method as proposed in this paper combines spatial and temporal unwrapping techniques. Since the orders solution of fringe with complete period is based on the mapping relationship between the frequency of the coded image and the fringe order, it is classified as a temporal unwrapping technique. Differently, the orders solution for noncomplete period fringe is based on continuity of the phase in the direction of fringe projection and consistency of phase in the direction of fringe, so that it is categorized into spatial unwrapping technique. Since the solution of the fringe order for the complete period plays a vital role in achieving phase demodulation, however, it is preferable to mention the method proposed as a temporal unwrapping technique. Relative to other spatial unwrapping techniques, the proposed method is advantageous in some respects. On the one hand, the implementation of the proposed method is not completely reliant on the continuity of the phase, but mainly on the frequency of the coded fringe, which means that the environment and the surface characteristics of the object have no impact. As a result, it shows robustness to external interference. On the other hand, the sampling frequency of FFT is determined according to the phase interval of the wrapped phase, which can be adjusted depending on the surface variation of the object. Compared with other temporal unwrapping techniques, the proposed method shows the following advantages. On the one hand, when the same number of fringe orders is demodulated, the number of coded patterns needing to be projected may be less than if other methods were applied, as shown in Table 2. On the other hand, since the two coded fringes are decoded at the same interval as the wrapped phase, the phase jump error of 2π is eliminated from the absolute phase as demodulated using the proposed method.

**Figure 22:**
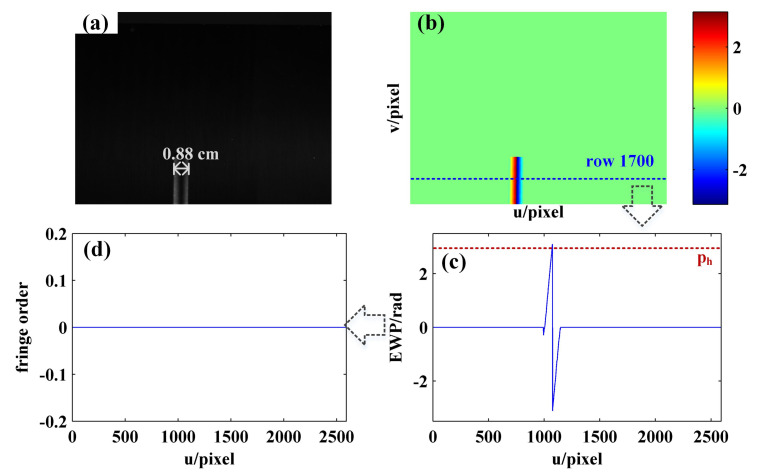
Order decoding results of the cuboid: (**a**) the sinusoidal fringe image with initial phase of −π acquired by camera; (**b**) EWP map; (**c**) EWP curve of row 1700; (**d**) fringe order of row 1700.

According to the orders decoding process as introduced in Figure 3 and Section 3.2, it can be found out that the core idea of the proposed phase unwrapping method is to perform orders decoding for complete periodic fringes; then, it is achieved for all fringes by combining the consistency and continuity of the fringe orders. As a result, it is possible for the proposed method to fail in the absence of sinusoidal fringe with complete period on the surface of the measured object. For example, Figure 22a shows a sinusoidal fringe image of a cuboid surface with the width of 0.88 cm acquired by the camera. According to the calculated EWP shown in Figure 22b,c, it can be seen that the number of complete periods on the object surface is less than 1 (i.e., the number of phase peaks whose phase is greater than $p_{h}$ is less than 2), which makes it impossible to realize the phase unwrapping of the object surface using the proposed method, as shown in Figure 22d.

To sum up, when phase unwrapping is carried out using the method proposed in this paper, the number of complete sinusoidal fringes on the object surface acquired by the camera should be greater than 1, and the correct fringe order decoding result can be obtained.

## Figures and Tables

**Table 1:** The average running time of proposed method and three-frequency phase-shift method.

Methods	Sphere	Three-Blade Fan	Step Workpiece
**three-frequency phase-shift method**	13.533 *s*	15.118 *s*	13.418 *s*
**proposed method**	22.0626 *s*	25.216 *s*	20.594 *s*

**Table 2:** The comparison result of the number of additional coded patterns required in addition to sinusoidal fringe patterns when phase unwrapping is performed using the multifrequency phase-shift method, gray coded method, and the proposed method.

Methods	Minimum Number	To Decode 10 Fringe Orders	To Decode 100 Fringe Orders	To Decode 1000 Fringe Orders
**multi-frequency phase-shift method**	3	3,6,9, …	3,6,9, …	6,9,12, …
**Gray coded method**	1	4	7	10
**proposed method**	1	2	3	4

## Data Availability

The raw/processed data required to reproduce these findings cannot beshared at this time as the data also forms part of an ongoing study.
